# SCF Ligases and Their Functions in Oogenesis and Embryogenesis—Summary of the Most Important Findings throughout the Animal Kingdom

**DOI:** 10.3390/cells11020234

**Published:** 2022-01-11

**Authors:** Veronika Kinterová, Jiří Kaňka, Alexandra Bartková, Tereza Toralová

**Affiliations:** 1 Laboratory of Developmental Biology, Institute of Animal Physiology and Genetics of the Czech Academy of Sciences, 27721 Libechov, Czech Republic; Kanka@iapg.cas.cz (J.K.); abartkova2@ukf.sk (A.B.); toralova@iapg.cas.cz (T.T.); 2 Faculty of Natural Sciences, Constantine the Philosopher University in Nitra, 949 01 Nitra, Slovakia

**Keywords:** ubiquitin-proteasome system, ubiquitin, SCF ligases, oogenesis, embryogenesis

## Abstract

SCF-dependent proteolysis was first discovered via genetic screening of budding yeast almost 25 years ago. In recent years, more and more functions of SCF (Skp1-Cullin 1-F-box) ligases have been described, and we can expect the number of studies on this topic to increase. SCF ligases, which are E3 ubiquitin multi-protein enzymes, catalyse protein ubiquitination and thus allow protein degradation mediated by the 26S proteasome. They play a crucial role in the degradation of cell cycle regulators, regulation of the DNA repair and centrosome cycle and play an important role in several diseases. SCF ligases seem to be needed during all phases of development, from oocyte formation through fertilization, activation of the embryonic genome to embryo implantation. In this review, we summarize known data on SCF ligase-mediated degradation during oogenesis and embryogenesis. In particular, SCF^βTrCP^ and SCF^SEL-10/FBXW7^ are among the most important and best researched ligases during early development. SCF^βTrCP^ is crucial for the oogenesis of *Xenopus* and mouse and also in *Xenopus* and *Drosophila* embryogenesis. SCF^SEL-10/FBXW7^ participates in the degradation of several RNA-binding proteins and thereby affects the regulation of gene expression during the meiosis of *C. elegans*. Nevertheless, a large number of SCF ligases that are primarily involved in embryogenesis remain to be elucidated.

## 1. Introduction

Mammalian oogenesis and embryogenesis are extremely important processes. Primordial germ cells are converted into oogonia and subsequently to oocytes, and the diploid cell is transformed into a haploid oocyte ready for fertilization. The oocyte grows and accumulates organelles, mRNA and proteins, which are used during the first embryonic stages [1]. During oogenesis, the oocyte is arrested at the diplotene stage of prophase meiosis I in a structure called a germinal vesicle (GV). Just before ovulation, after the increase in gonadotropin hormones and activation of cyclin dependent kinase (CDK1, a catalytic subunit of the M phase-promoting factor MPF), meiosis is reactivated and the nuclear membrane breaks in a process called germinal vesicle breakdown (GVBD). Subsequently, cytokinesis occurs and the first polar body is extruded. The oocyte enters the second meiotic division and arrest in metaphase II (MII), where the oocyte remains until its fertilization [2]. Only matured oocytes are able to undergo fertilization. Contact with sperm causes intracellular calcium ion oscillation, leading to oocyte activation and meiosis initiation [3]. During fertilization, the haploid oocyte and haploid sperm fuse together to create a zygote, and the first mitotic division occurs within several hours [4]. Early preimplantation development is a highly complicated and strictly regulated process. Preimplantation development starts with several rapid cell cycles with short or completely lacking G phases [5]. These early stages of development are controlled by maternal mRNAs and proteins accumulated during oogenesis. Subsequently, the control of development passes from maternal to embryonic in a process called the maternal-to-zygotic transition (MZT), leading to embryonic genome activation (EGA) [6]. A minor wave of EGA shortly after fertilization is followed by a more robust major wave later in development. The first transcripts were found only 7 h after pronuclei formation in mice [7]. The latest studies suggest that embryonic genome activation is a gradual process that appear with smaller waves of transcription. For example, splicing factor arginine/serine-rich 3 (SRFS3) was found to be already expressed at the 4-cell stage, thus during the minor genome activation in cattle [8]. Maternal reserves are gradually replaced by their embryonic forms and removed from the embryo. Maternal mRNAs are presumably degraded by miRNAs and probably also by other classes of small non-coding RNAs in early embryos [9]. One of the potential pathways of maternal protein degradation is via the ubiquitin-proteasome system (UPS). UPS plays a crucial role in many steps of gametogenesis and embryogenesis.

**Figure 1:**
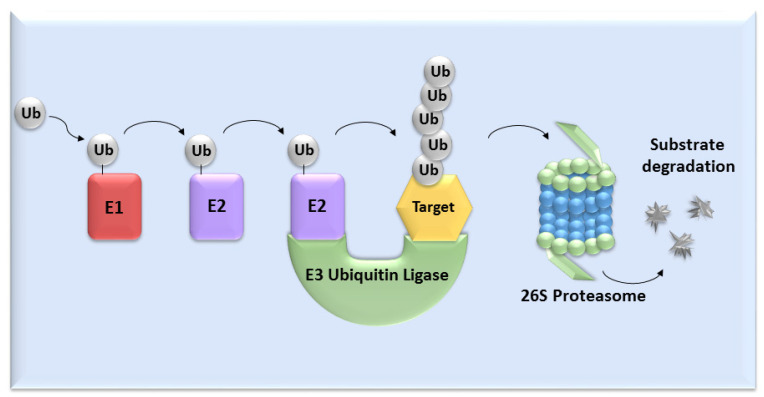
The ubiquitin-proteasome system. Activation and binding of ubiquitin to the target substrate is mediated by three enzymatic complexes (E1, E2 and E3 enzyme). The substrate is selected by E3 ubiquitin ligase and after tagging by ubiquitin, substrate undergoes degradation mediated by 26S proteasome.

**Figure 2:**
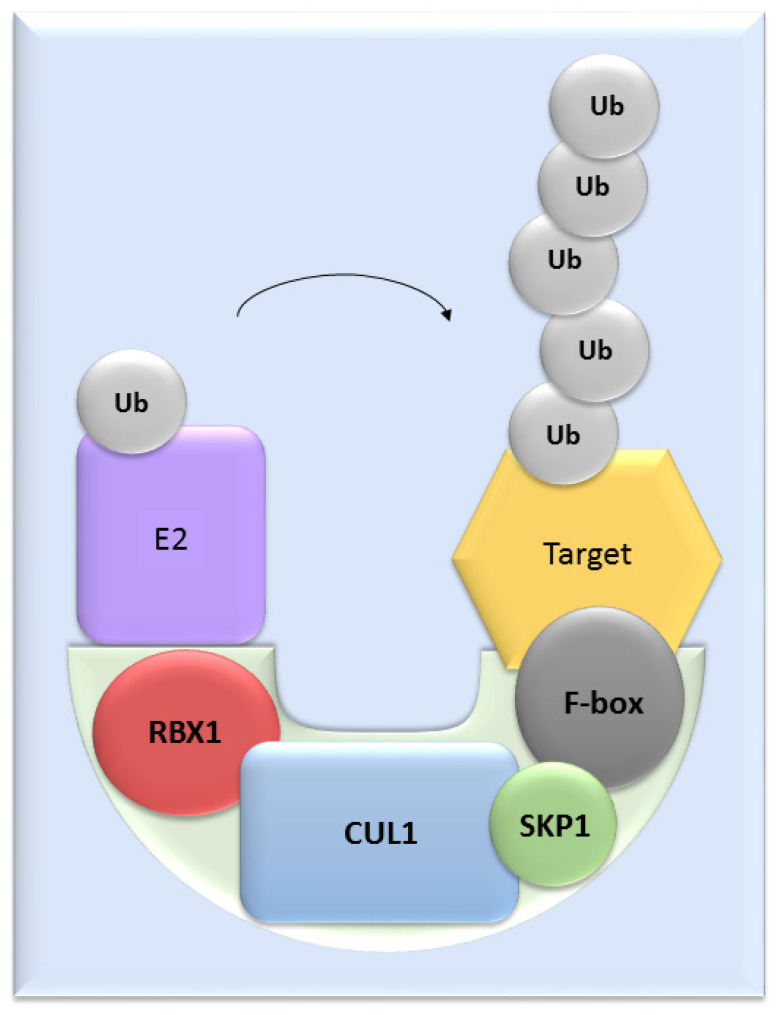
The structure of SCF ligases. One of the most common ligase is composed by RBX1, CUL1, SKP1 protein and a member from the F-box protein family.

UPS mediates the proteolysis of a variety of proteins that are important for many basic cellular processes, which include the regulation of cell cycle and development, response to stress, DNA repair, modulation of surface receptors and channels, regulation of transcription and many others. This wide range of processes is due to the large number of individual enzymes that belong to the UPS, which affect a huge number of target substrates. The degradation of proteins by the UPS is based on labelling the targeted protein with ubiquitin. This process is called ubiquitination and involves three enzymatic complexes: E1 ubiquitin-activating enzyme, E2 ubiquitin-conjugating enzyme and E3 ubiquitin ligases (Figure 1). The E3 enzyme mediates the final interaction between ubiquitin and the substrate, and hence is responsible for substrate specificity. Proteins are polyubiquitinated (labelled with more than just one molecule of ubiquitin) and subsequently directed to degradation by the 26S proteasome [10]. Based on the presence of characteristic domains and mechanisms of ubiquitin transfer from E2 enzyme to the specific substrate proteins, E3 ligases are divided to the three main groups: RING (really interesting new gene) E3s, HECT (homologous to the E6AP carboxyl terminus) E3s and RBR (RING-betweenRING-RING) E3s [11]. One of the most abundant and common RING E3 enzymes is SCF (Skp1-Cullin 1-F-box) ligase, composed of three invariant members: cullin1, SKP1, RBX1 and one of the F-box proteins, which determines the substrate specificity (Figure 2). Besides Cul1-based SCF ligases, there are also other types of SCF ligases formed by different cullins (Cul2-9). These complexes are called CRLs (Cullin-RING ubiquitin ligases, followed by the number of the cullin) or sometimes known as Cullin 2-9-based SCF complexes. In SCF complexes, the F-box protein determines the substrate specificity [12]. A large number of F-box proteins provide interaction with a wide range of substrates, and in this way SCF ligases are involved in the regulation of many processes, including the regulation of gametogenesis and embryonic development.

## 2. SCF Ligases Composition

### 2.1. Cullins

In mammals, seven different cullins are found: Cul1, Cul2, Cul3, Cul4A, Cul4B, Cul5 and Cul7 [12]. The activation of cullins is mediated by neddylation (modification with the protein Nedd8) [13]. After deneddylation, Cul binds to CAND1 (Cullin-associated and neddylation dissociated 1) and inactivates the SCF ligase [14].

In cattle, Cullin1 is expressed from two different genes. The first type is expressed from the MII oocyte until the early 8-cell stage and is called cullin1-like or maternal Cul1. The second one is expressed from the 8-cell stage to the blastocyst stage and is called Cul1 or embryonic Cul1. Both share 83% homology and in somatic cells, the embryonic form of Cul1 is expressed [15]. The expression of Cul1-like mRNA is relatively high from the MII to the 4-cell stage, but at the early 8-cell stage, the mRNA level rapidly decreases and stays low until the blastocyst stage. In contrast, Cul1 mRNA level is low from the MII to the 4-cell stage and gradually increases from the early 8-cell stage, with the highest level at the blastocyst stage [16].

It was recently found that CUL1 is upregulated in lamb oocytes after IVM. This suggests earlier maternal protein degradation, which could contribute to the lower developmental competence of oocytes [17].

The deletion of Cul1 in mice embryos caused arrest at E6.5 before the onset of gastrulation. A high level of cyclin E protein was found in these embryos, suggesting that cyclin E abundance is controlled by SCF ligase activity [18,19].

In mice, CUL1 also plays an important role in later embryonic development. CUL1 was detected in trophoblast cells at E9.5 and spongiotrophoblast at E11.5 and E13.5. After CUL1 knockout embryos failed to implant, indicating that a decreased expression of CUL1 affects the placentation of mice [20]. The necessity of CUL1 for trophoblast proliferation, implantation and placental development was also confirmed also by Sun et al. [21], who observed the role of CUL1 in human trophoblast cells and its connection to recurrent spontaneous abortion (RSA). In all RSA samples, the levels of neddylated cullins (especially neddylated-Cul1) were downregulated, leading to the accumulation of p21 [21]. Furthermore, Zhang et al. [22] discovered that CUL3 has an essential role in the invasion and migration of trophoblast cells, and its dysregulation may be associated with the onset of pre-eclampsia. The necessity of cullins in later embryogenesis was also confirmed by Tsunematsu et al. [23]. They found that the CUL1-FBXW8-CUL7 complex plays an important role in the mid- to late stage of development of the placenta, because Fbxw8^−^ mouse embryos failed to develop a functioning placenta [23].

### 2.2. RBX1 (Ring-Box 1)

Rbx1 mediates cullin neddylation and contains a RING finger domain recruiting the E2 enzyme [24]. Rbx1, attached to Cul, forms a catalytic core of SCF ligases and, based on the type of connected cullin, activates several types of SCF ligases [25]. The expression of Rbx1 starts at the time of a major wave of EGA in cattle, at the late 8-cell stage [16]. The silencing of Rbx1 caused embryonic lethality at E7.5 in mouse embryos. This developmental arrest was caused by an accumulation of p27, leading to hypoproliferation [26]. Moreover, the silencing of Rbx1 by RNAi causes defects in the first meiotic division of oocytes and in mitotic chromosomal condensation and segregation, abnormal cortical protrusion, multinucleate cells and defects in germ cell proliferation of *Caenorhabditis elegans* embryos [27]. The necessity of Rbx1 was also verified by Jia et al. [28], who confirmed that the silencing of Rbx1 induced embryonic lethality of *C. elegans*. Jia et al. [28] presumed that this could be caused by an accumulation of a number of substrates of SCF ligases, because SCF ligases control the turnover of many short-lived regulatory proteins in a cell content-dependent and spatially dependent manner. Rbx1, namely its homolog called Roc1a, is also required for the cell proliferation and embryonic development of *Drosophila melanogaster* [29].

### 2.3. SKP1 (S-Phase Kinase-Associated Protein 1)

Skp1 mediates the binding of the F-box protein to Cul1 [30]. The dimerization of Skp1 causes overlaps of the F-box protein binding site, leading to the prevention of Skp1 and F-box protein interaction [31]. The mRNA transcription of bovine Skp1 starts at the early 8-cell stage, indicating an important role during the EGA that occurs at the late 8-cell stage in cattle [16]. Skp1 also plays an essential role in the prophase I to metaphase I transition during sperm development. SKP1-deficient spermatocytes exhibit precocious pachytene exit, premature desynapsis, loss of PLK1 and BUB1 at centromeres with a persistence of HORMAD, γH2AX, RPA2 and MLH1 in diplonema and reduced MPF activity [32]. Changes in Skp1 expression play a role in several diseases, for example in the development of Parkinson’s disease [33] and lymphomas [34].

### 2.4. F-Box Proteins

A large number of mammalian F-box proteins have been identified so far. F-box proteins are divided into three subgroups based on their structural conformation: FBLs, FBXs and FBWx. FBW proteins contain WD40-repeat domains, FBL proteins contain leucine-rich repeats and FBX contain other structures [35]. Based on the existence of these motifs, F-box proteins are able to connect a wide range of substrates to the SCF complex. F-box proteins are short-lived proteins regulated by auto-ubiquitination. The result of their auto-ubiquitination is a rapid switching among multiple SCF complexes leading to cells adaptation to changing physiological conditions and progression through individual steps of the cell cycle [36].

## 3. SCF Ligases in Oocyte Maturation

**Figure 3:**
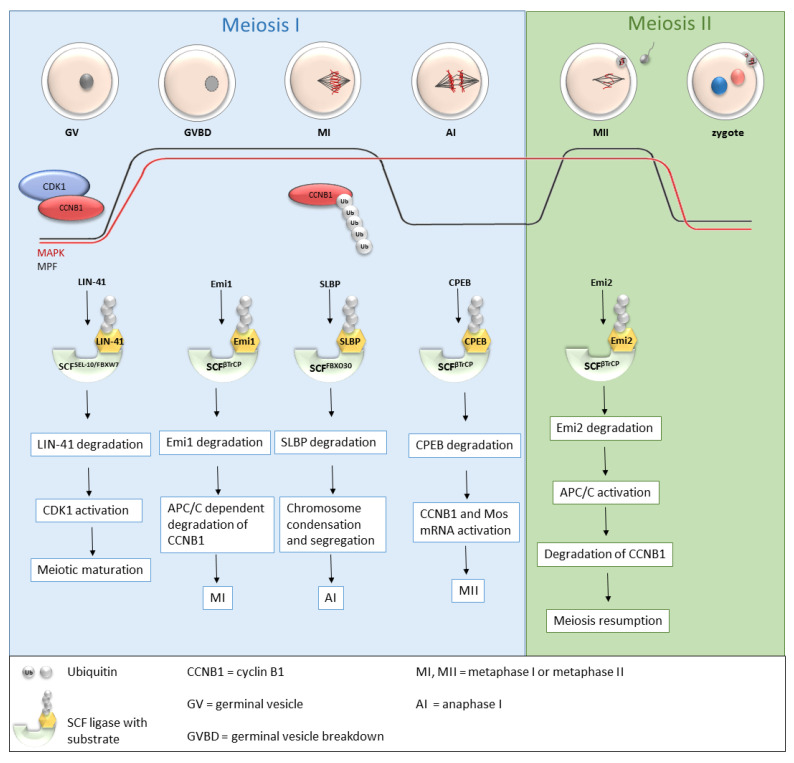
Roles of selected SCF ligases in oogenesis. Progression through oogenesis is mediated by MAPK and MPF (composed by CDK1 and CCNB1) activity which is graphically represented by red and black curves. Activation of CDK1 is regulated by a cyclic activity of CCNB1, whose degradation is mediated via ubiquitination by APC/C. The level of MAPK is stable to the metaphase II. SCF^SEL-10/FBXW7^, SCF^βTrCP^ and SCFFBXO30-mediated degradation of their targets is essential for progression through meiosis I and meiosis II stages of mouse, C. elegans and Xenopus oocytes. AI—anaphase I, APC/C—anaphase promoting complex/cyclosome, CCNB1—cyclin B1, CDK1—cyclin-dependent kinases I, CPEB—cytoplasmic polyadenylation element-binding protein, Emi1, Emi2—early mitotic inhibitor 1 or 2, GV—germinal vesicle, GVBD—germinal vesicle breakdown, MI, MII—metaphase I, metaphase II, MAPK—mitogen-activated protein kinase, MPF—M phase-promoting factor, SCF—Skp1-Cul1-F-box complex, SLBP—stem-loop-binding protein, Ub—ubiquitin.

Recently, more and more studies have been dealing with the role of SCF ligases during oogenesis (Table 1 and Figure 3), and the roles of several SCF ligases have been proven, predominantly in lower organisms [37] or mice [37,38,39]. During oogenesis, oocytes are initially arrested in the prophase of the first meiotic division with a low MPF level. GVBD and progression into metaphase I is mediated by an increasing level of MPF, leading to the first polar body extrusion. Immediately after GVBD, early mitotic inhibitor 1 (Emi1), an inhibitor of anaphase-promoting complex/cyclosome (APC/C), undergoes SCF^βTrCP^-dependent degradation [40,41]. The degradation of Emi1 is stimulated by phosphorylation and βTrCP binding mediated by Plk1 [42]. APC/C is responsible for cyclin B destruction and thereby inactivation of MPF. Thus, the degradation of Emi1 leads to APC/C activation, subsequent degradation of cyclin B and MPF activation [40,41]. From the MI stage, oocytes continue immediately to the second meiosis. The meiotic arrest of oocytes at the MII stage is driven by the stabilization of the high activity of MPF by cytostatic factor (CSF). CSF inhibits APC/C and thereby prevents the degradation of cyclin B. Another of the CSF components is Emi2, a direct inhibitor of APC/C [43].

Emi2 (also named Erp1, human and mouse orthologs are called FBXO43 [44]) mediates MII arrest by inhibiting the binding of Ube2S to the APC/C in *Xenopus laevis* eggs. Ube2 is an ubiquitin-conjugating enzyme which cooperates with APC/C in APC/C-dependent protein degradation. Ube2S is important for cyclin B1 and B2 degradation and thus for the subsequent nuclear envelope formation during cytostatic factor release. The Emi2-dependent inhibition of APC/C prevents the release of CSF. CSF blocks metaphase by the inhibition of APC/C (by Emi1) and subsequent stabilization of cyclin B. During CSF release (or fertilization, respectively), the degradation of Emi2 relieves inhibition of the APC/C-Ube2s binding, leading to rapid cyclin B1 and B2 degradation [45]. Emi2 is degraded by SCF^βTrCP^ in response to calcium signalling [44].

SCF^βTrCP^ is also important for the CPEB (cytoplasmic polyadenylation element-binding protein) degradation during *Xenopus* oocyte maturation. βTrCP binds to the highly conserved sequence _190_TSGFSS_195_ of CPEB if phosphorylated at certain amino acids (T190, S191 and S195). Phosphorylation at S191 of CPEB is mediated by PLK1, at T121 by Cdc2, and thereby creates a docking site for PLK1 [46]. PLK1 is known to phosphorylate several regulators of the cell cycle: Emi1 in mitosis [51], somatic Wee1 [52] and Erp1 in *Xenopus* meiosis [53] and targets them for degradation by SCF^βTrCP^. The degradation of CPEB is necessary for the activation of some mRNAs, for example mRNA encoding cyclin B1 and Erp1, thereby driving the oocyte to entry into meiosis II [54].

In contrast, protection from SCF^βTrCP1^-mediated ubiquitination was demonstrated to be important for the MZT and maternal mRNA clearance in mice. Nuclear Poly(A)-binding proteins (PABPNs) are exclusively involved in mRNA post-transcriptional regulation. A maternal form of nuclear Poly(A)-binding protein called PABPN1L acts as an mRNA-binding adapter of BTG4 and thereby prevents the degradation of BTG4 by SCF^βTrCP1^. BTG4 mediates a maternal mRNA clearance and thus the maintenance of BTG4 creates an expression window for MZT-coupled maternal mRNA decay in maturing mice oocytes. PABPN1L is expressed exclusively after meiotic resumption, with a maximal level at the MII stage. Maternal PABPN1L knockout causes an embryonic arrest at the 1- to 2- cell stage with the inability to develop further, leading to infertility of these knockout mice. These findings suggest the necessity of PABPN1L for the MZT and also the importance of SCF ligases for embryonic genome activation of mammals [47].

During the meiotic prophase of *C. elegans*, two distinct RNA-binding proteins (RBPs), key regulators of gene expression, are synchronously degraded by SCF^SEL-10^ [37]. Hyperphosphorylation of both these RBPs (GLD-1 and CPB-3) caused by MAPK signalling activity leads to their tagging for recognition by F-box protein. Kisielnicka et al. [37] demonstrated that SEL-10/FBXW7 (SEL-10 is homologous to mammalian FBXW7; Fbxw7 is also known as Cdc4) and MPK-1/MAPK bind to CPB-3 and GLD-1 in heterologous systems. Therefore the authors suggest that MAPK and FBXW7 work together as a regulatory pathway in meiotic progression which is synchronized with the posttranscriptional gene expression of gametogenesis. SEL-10 works as a substrate recognition subunit that mediates the ubiquitination and subsequently destabilization of GLD-1 and CPB-3 in postpachytene oocytes. In mammalian somatic cells, FBXW7 participates as a negative regulator of cyclin E, Notch and the two proto-oncogenes c-Myc and c-Jun [55]. While Fbxw7 is an essential gene for mouse embryonic development, Sel-10 is not essential for *C. elegans* [37]. Fbxw7 also causes the degradation of CDC6 protein. CDC6, being a protein important for the initiation of DNA replication, spindle formation and the regulation of M-phase progression by activation checkpoint mechanisms and by inhibiting Cdk1 activity during mitosis exit, has to be precisely regulated to avoid the inhibition of Cdk1 in *Xenopus* oocytes. The precise degradation of CDC6 shortly before entry into meiosis I is provided by SCF^Fbw7^ and the proteasome. Its degradation is controlled by the initial activation of Cdk1 [48].

Furthermore, SCF ligases regulate the fate of another RNA-binding protein called LIN-41. LIN-41 is a translational repressor that controls oocyte growth and meiotic maturation by preventing premature activation of cyclin-dependent kinase CDK1 in *C. elegans* [56]. After the beginning of meiotic maturation, LIN-41 is rapidly eliminated from the oocyte. Its elimination requires the activities of CDK1 and multiple types of SCF ligases. CDK1 is reciprocally inhibited by LIN41. After the activation of CDK1, meiotic maturation is stimulated and the SCF-dependent degradation of LIN41 is promoted. The substrate adaptor F-box protein participating in LIN-41 degradation is called SEL-10/Fbw7(human)/Cdc4 (yeast) [49]. Spike et al. [49] determined that LIN-41 must be inactivated before its degradation by SCF^SEL-10^ E3 ubiquitin ligase by the end of the first meiotic division. LIN-41 can most likely also play a role in shaping the proteome during the maternal-to-embryonic transition. They also found that the protein GLD-1 (a tumour suppressor) undergoes a similar regulation process to LIN-41 [49]. GLD-1 controls and coordinates oocyte differentiation and cell cycle progression and is abundantly expressed through the pachytene stage but subsequently falls to the background level as the oocyte exits the pachytene stage and completes oogenesis. The expression of GLD-1 corresponds to its role in the oocyte, which may be the translational repression of part of the maternal RNAs synthesized during oogenesis [57].

Jin et al. [38] investigated the role of FBXO30 during mice meiosis by its deletion using siRNA microinjections. FBXO30 RNAi oocytes were able to undergo GVBD, however they were unable to exclude the polar body, and homologous chromosomes did not separate, which caused meiotic arrest. Moreover, an abnormal spindle assembly appeared at the Pro-MI stage, probably caused by an overcondensation of chromosomes leading to an inability of chromosomes to form the spindle assembly in a normal manner [38]. Furthermore, the potential substrate of FBXO30 was determined by comparing the RNAi oocytes with the control group using MS analysis, leading to the identification of stem-loop-binding protein (SLBP). An ineffective degradation of SLBP leads to an overload of histone H3 on chromosomes, leading to the overcondensation of chromosomes and segregation inhibition [38]. FBXO30 is also involved in embryonic development by controlling RARγ (retinoic acid receptor) levels by FBXO30-mediated ubiquitination and is also a key regulator of BMP (bone morphogenetic protein) signalling [58].

Zhao et al. [39] observed the function of FBXO34, another protein from the F-box family, during mouse oocyte maturation. They found that FBXO34 plays pivotal roles in oocyte maturation. The depletion of FBXO34 led to the failure of oocyte meiotic resumption caused by a low activity of maturation promoting factor (MPF). However, this phenotype could be rescued by the microinjection of exogenous CCNB1 (also known as cyclin B1), a regulatory subunit of MPF. This confirmed that the meiosis arrest at prophase I was caused by the low expression level of CCNB1 [39]. Increased level of CCNB1 and the dephosphorylation of CDK1 leads to MPF activation and the resumption of meiosis [59]. The overexpression of FBXO34 promoted GVBD (germinal vesicle breakdown), nevertheless these oocytes were arrested at the MI stage with stagnated chromosome separation and the absence of spindle migration to the cortex, leading to the failure of anaphase entry. This could be caused by an increased level of ubiquitination, leading to the degradation of one or more proteins and the impairment of meiosis progression [39]. However, the direct substrate of FBXO34 has not been found yet.

Some oocyte-specific F-box proteins that only appear during oogenesis were found. Chesnaye et al. [50] identified an F-box protein-encoding gene that is selectively expressed in oocytes of the mouse ovary. The predicted protein is termed FBXW15 or FBXO12J, and is a member of the F-box-only (FBXO) family. The continuously increased Fbxw15/Fbxo12 mRNA level likely helps to prevent oocytes from exiting meiotic prophase I and reaching the diplotene stage. This function is however ascribed to other ligases, such as RFPL4. Another option is that FBXW15/FBXO12J might function as a regulator of oocyte-granulosa communication [50]. Wang et al. [60] reported the expression analysis of an oocyte-specific gene coding a novel F-box protein in rainbow trout. They observed the Fbxoo mRNA and protein expression profiles and found high expression in oocytes at the early pre-vitellogenesis stage. This finding suggests an essential role of this F-box protein in early oocyte development by regulating other critical proteins [60].

## 4. SCF Ligases in Embryogenesis

Recently, the role of SCF ligases in maternal protein degradation has been frequently discussed [61,62,63,64]. Based on the expression of SCF complex members (Cul1, Skp1, Rbx1) during all stages of preimplantation development, it is presumed that the SCF complex plays an important role during the early embryogenesis of cattle [16,65]. The inhibition of SCF ligases by cultivation in MLN4924 (a specific inhibitor of cullin neddylation) in cattle leads to a statistically significant delay in development and also to a significant increase in the total protein level, although no accumulation of a specific protein has been found yet. Reduced levels of mRNA of EGA markers (PAPOLA, and U2AF1A) were found after MLN4924 treatment [63]. The importance of SCF ligases for oogenesis and embryogenesis is also assumed in mice, since a large number of F-box proteins have been found in oocytes and zygotes. Wang et al. [66] have identified 19 different F-box proteins overexpressed in the mouse oocytes, and presume that these F-box proteins play an important role in protein degradation after fertilization [66]. F-box proteins are also highly abundant in two-cell embryos [67]. The list of SCF ligases and their known substrates important for embryogenesis can be found in Table 2.

**Figure 4:**
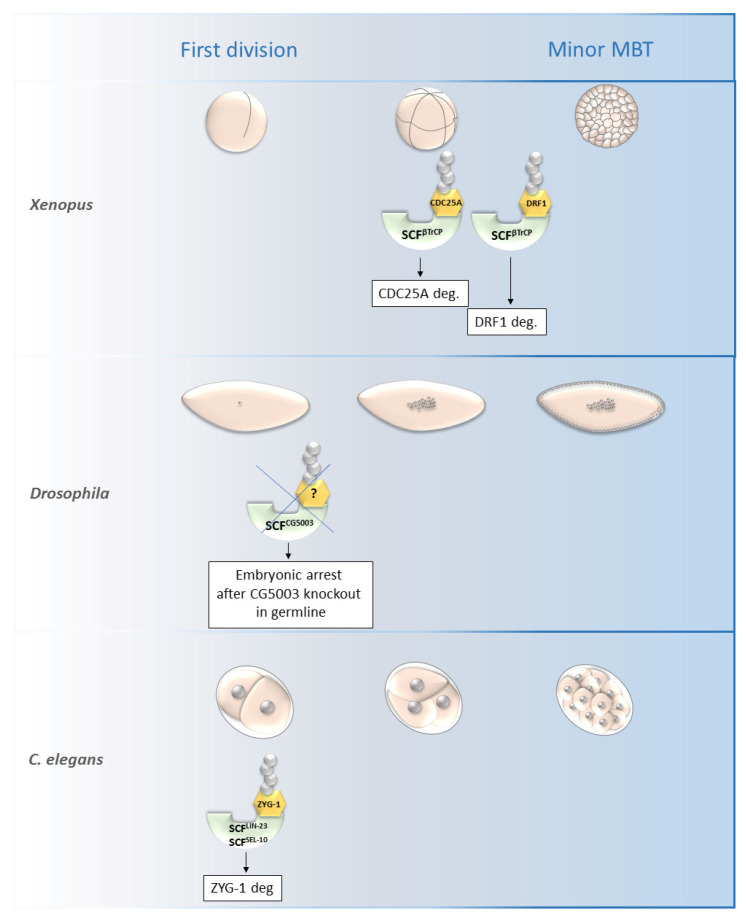
Roles of selected SCF ligases in embryogenesis. Figure shows selected SCF ligases and their roles in degradation of proteins during embryogenesis of *Xenopus laevis, Drosophila melanogaster* and *C. elegans*. The main impact of these selected ligases was observed from the first division until the minor MBT. The SCF^βTrCP^ and SCF^ZYG-1^- mediated degradation is important for early embryonic development of *Xenopus* and *C. elegans,* respectively. The specific substrate of SCF^CG5003^ in *Drosophila* development has not been described yet but its degradation via CG5003 is necessary for progression through the first and second embryonic division. MBT—mid-blastula transition, SCF—Skp1-Cul1-F-box complex, ZYG-1—zygote defective protein, CDC25A—M-phase inducer phosphatase 1.

SCF^β-TrCP^ was found to be necessary for the mid-blastula transition (MBT) in *Xenopus* as it is responsible for the degradation of DRF1 (a homolog of mammalian DBF4B) (Figure 4). DRF1 is a limiting replication initiation factor, whose degradation causes lengthening of the cell cycle after the MBT. The degradation of DRF1 is a result of developmental activation of the Chk1 (checkpoint kinase) during the MBT. It is the primary mechanism by which cell-cycle progression is blocked by Chk1 in the early embryo [62]. The constitutively active form of Chk1 phosphorylates CDC25A and targets it for subsequent degradation. CDC25A is another known protein degraded by SCF^β-TrCP^ at around the MBT of *Xenopus* embryos [61]. It is important for mitosis progression and also for the resumption of meiosis during mouse oocyte maturation [73]. Some another maternal proteins that need to be degraded for embryonic genome activation were found—CDC6, Treslin, RECQL4, TOPBP1 in *Xenopus* [74], but to the best of our knowledge, the precise mechanism of their degradation has not been specified yet.

Significantly fewer proteins degraded at the time of EGA have been found in mammals. In mice, TAB1 degradation is mediated by a non-SCF E3 ligase called Ring finger protein 114 (RNF114) and is needed for a normal course of EGA [75]. Degradation of the maternal protein PIASy (protein inhibitor of activated STATy), E3 SUMO ligase, seems to be important for proper EGA. The overexpression of PIASy caused the developmental arrest of mouse embryos at the 2-cell stage. Increased trimethylation of histone H3 lysine 9 (H3K9me3) in 2-cell nuclei and also increased translocation of H3K9me3 methyltransferase to the pronucleus were found in these embryos. The degradation of PIASy is mediated by UPS, but the specific E3 ligase mediating its ubiquitination has not been reported yet [76].

Another protein which is degraded by SCF ligases is the kinase PLK4. It is required for centrosome duplication and is regulated by SCF^Slimb/βTrCP^ in human and *Drosophila* cells. ZYG-1, a *C. elegans* homolog of PLK4, is regulated by a similar mechanism—using the βTrCP homolog LIN-23 and F-box protein SEL-10 (Figure 4) [68,70]. SEL-10 is a conserved protein with homologs in humans (FBW7) and also in *Drosophila* (archiplelago; ago). Both these proteins are implicated in the degradation of cyclin E, however the suppression of ZYG-1 by the reducing activity of SEL-10 does not lead to an elevation of the level of cyclin E [70]. Slimb also fulfils several role in embryonic development. Muzzopappa and Wappner [69] observed the expression of this protein during *Drosophila* embryogenesis and found it to be important for egg chamber development and the differentiation of follicular cells. They also presumed that Medea (a *Drosophila* homolog of the mammalian SMAD protein) can be targeted by Slimb for degradation, because SMAD is degraded by βTrCP1 in mammals [69].

SCF ligases are also important for the embryogenesis of zebrafish. Smad ubiquitination regulatory factor 1 (Smurf1), a homolog of E6AP C-terminus (HECT)-type E3 ubiquitin ligase is negatively regulated by SCF^FBXL15^ ligase in zebrafish embryos. Knockout of FBXL15 caused BMP deficiency and a developmental defect. Smurf1 is a crucial regulator of the bone morphogenetic protein (BMP) pathway, and FBXL15 depletion leads to BMP-deficient embryos [71].

Avilés-Pagán et al. [72] identified an uncharacterized gene (CG5003) that encodes an F-box protein, and found that mutation of this gene causes a decrease in female fertility and early embryonic arrest in *Drosophila* embryos. A significant proportion of embryos arrested or delayed during the first or second mitotic divisions after the knockout of CG5003 in the germline (Figure 4), although no change in phenotype was detected during oogenesis. These results suggest the existence of an SCF^CG5003^ complex with specific targets destined to be degraded, and whose degradation is allowed to progress though the first few rounds of embryonic divisions. However, since not all CG5003-null embryos arrest, the authors presume that other SCF complexes or different E3 ubiquitin ligases can substitute for the functions of SCFC^G5003^ at this point. So each SCF complex might have a preference among their substrates and not focus purely on the specific ones whose degradation must be mediated during embryogenesis [72]. However no specific substrate of CG5003 F-box protein has been described yet.

## 5. Conclusions/Perspectives

In this review, we summarized current knowledge about the functions of SCF ligases in oogenesis and early embryogenesis. SCF^β-TrCP^ is one of the best examined ligases in oogenesis and embryogenesis with the most described substrates and functions. It has been demonstrated that SCF^β-TrCP^ is involved in the regulation of oocyte maturation by the degradation of CPEB, Emi1 and Emi2 [40,45,46]. Their degradation is necessary to overcome meiosis progression and meiosis arrest. During embryogenesis, SCF^β-TrCP^ plays a pivotal role in the degradation of some maternal proteins, such as DRF1 and CDC25A in *Xenopus* embryos [61,62]. On the other hand, the protection of BTG4 from β-TrCP-mediated degradation is essential for proper maternal mRNA clearance during mouse development [47]. Another well-known ligase is SCF^SEL-10/FBXW7^, which regulates the degradation of several RNA-binding proteins during *C. elegans* oogenesis. These RNA-binding proteins are GLD-1, CPB-3 and LIN-41, which participate in the regulation of gene expression and thus control the progression of meiosis [37,49]. SCF^FBXW7^ also mediates the degradation of CDC6 protein, which is important for DNA replication, spindle formation and M-phase progression in *Xenopus* oocytes [48]. Other members of the F-box family have been identified as having functions in oogenesis and embryogenesis but their substrates have not been discovered yet.

The described roles of SCF ligases in meiosis control, chromosome segregation and developmental progression can potentially lead to a better understanding of problems in human assisted reproduction. In the coming years, we can expect more and more studies to describe new functions of SCF ligases. The balance of protein formation and degradation is obviously key for the proper regulation of cell cycle progression, meiosis and also for the early embryonic development of vertebrates.

## Figures and Tables

**Table 1:** Known SCF ligases and their substrates in oogenesis of mouse, *Xenopus* and *C. elegans* oocytes.

SCF Ligase	Substrates	Animal	Reference
SCF^βTrCP^	Emi2/Erp1	*Xenopus laevis*	[44]
	Emi1		[45]
	CPEB		[46]
	BTG4	Mouse	[47]
SCF^SEL-10/FBXW7/CDC4^	GLD-1,CPB-3	*Caenorhabditis elegans*	[37]
	CDC6		[48]
	LIN-41		[49]
SCF^FBXO30^	SLBP	Mouse	[38]
SCF^FBXO34^	Not identified	Mouse	[39]
SCF^FBXW15,FBXO12J^	Not identified	Mouse	[50]

**Table 2:** Known SCF ligases and their substrates in embryogenesis of human, *Xenopus*, *C. elegans*, *Drosophila* and zebrafish embryos.

SCF Ligases and Their Substrates in Embryogenesis			
SCF Ligase	Substrates	Animal	Reference
**SCF^β-TrCP/Slimb/LIN-23^**	DRF1	*Xenopus laevis*	[62]
	CDC25A		[61]
	PLK4	*Drosophila melanogaster*	[68]
	Medea		[69]
	ZYG-1	*Caenorhabditis elegans*	[70]
**SCF^SEL-10/FBXW7/CDC4^**	ZYG-1	*Caenorhabditis elegans*	[70]
**SCF^FBXO30^**	RARγ, BMP	Human	[58]
**SCF^FBXL15^**	Smurf1, BMP	Zebrafish	[71]
**SCF^CG5003^**	Not identified	*Drosophila melanogaster*	[72]

## Data Availability

Not applicable.
